# Molecular-scale origins of wettability at petroleum–brine–carbonate interfaces

**DOI:** 10.1038/s41598-020-77393-4

**Published:** 2020-11-25

**Authors:** Paul Fenter, Tianzhu Qin, Sang Soo Lee, Mohammed B. AlOtaibi, Subhash Ayirala, Ali A. Yousef

**Affiliations:** 1 Chemical Sciences and Engineering Division, Argonne National Laboratory, Lemont, IL 60439 USA; 2 EXPEC Advanced Research Center, Saudi Aramco, Dhahran, Saudi Arabia

**Keywords:** Environmental sciences, Chemistry, Energy science and technology, Materials science

## Abstract

Wettability control of carbonates is a central concept for enhanced petroleum recovery, but a mechanistic understanding of the associated molecular-scale chemical processes remains unclear. We directly probe the interface of calcium carbonate (calcite) with natural petroleum oil, synthetic petroleum analogues, and aqueous brines to understand the molecular scale behavior at this interface. The calcite–petroleum interface structure is similar whether or not calcite was previously exposed to an aqueous brine, and is characterized by an adsorbed interfacial layer, significant structural changes within the calcite surface, and increased surface roughness. No evidence for an often-assumed thin-brine wetting layer at the calcite–petroleum interface is observed. These features differ from those observed at the calcite–brine interface, and for parallel measurements using model synthetic petroleum mixtures (consisting of representative components, including dodecane, toluene, and asphaltene). Changes to the interface after petroleum displacement by aqueous brines are also discussed.

## Introduction

Wettability is a central concept for understanding oil displacement from geological reservoirs^1^. It is normally assumed that pore fluid wetting is controlled by the presence of a thin brine film, located between the rock surface and the crude oil, whose behavior depends on the interactions at the brine–oil and rock–brine interfaces^2–4^. The extent of the proposed aqueous film thickness ranges from a few molecular layers to thicknesses of ~ 10 nm^5,6^. Increasing attention has been given to the chemical interactions at the rock–fluid interface and its control of wettability alteration in carbonate rocks during low-salinity water-flooding for enhanced oil recovery^7–9^. For example, measurements of surface forces and adhesion at mica and glass surfaces reveal that thin brine films are stabilized at salt concentrations below, and pH values above, critical values^2,3,10–12^. These ideas have been explained on the basis of the well-kjnown Derjaguin, Landau, Verwey, and Overbeek (DLVO) theory^10^.

The mechanisms to explain this behavior generally fall within two distinct concepts: the *double layer expansion* (DLE) model^13,14^ posits the presence of an aqueous wetting layer between the carbonate and petroleum phases, whose thickness is controlled by changes in the diffuse layer screening in low ionic strength brines. This concept implicitly assumes that the carbonate surface has a pH-dependent surface charge (e.g., due to protonation reactions of the surface functional groups), which is screened by counter ions, either adsorbed on the surface or distributed in a diffuse ion layer (with a screening length that is controlled by the ionic strength of a solution)^15–21^. This picture is well-established for oxide–water interfaces, such as the rutile (TiO_2_) (110) surface^22–24^. In contrast, molecular scale measurements of the well-defined calcite (CaCO_3_) (104) cleavage surface have found that its speciation does not follow this prediction^25^, and separate measurements of ion adsorption suggest that the net charge of a calcite surface is quite small^26^. This suggests that one of the conceptual foundations for the DLE model is not supported by direct observations of the intrinsic chemical reactivity of well-defined model systems.

In contrast, the *carbonate surface reactivity* model posits structural and compositional changes at the carbonate–petroleum interface within the internal pores of the rock due to a combination of carbonate dissolution and surface adsorption^27–30^. For example, it has been suggested that ionic aggregates in thin brine films act as anchors to hold oil components in the vicinity of the substrate, and that these anchors are disrupted in low salinity water in favor of the water-wet state^16,31^. These two mechanisms are clearly distinct, but have in common the implicit assumption of a reactive carbonate surface that provides sites for adsorption from the brine or the petroleum phase.

In spite of the wealth of information concerning the enhancement of oil displacement by saline waters, there are very few studies that have explicitly characterized, through in situ observations, the interfaces between rock surfaces with petroleum oil and brines at a level that provides a direct test of these ideas. Consequently, the appropriate conceptual model of the associated interfacial interactions has not been tested directly. The goal of this study, therefore, is to provide direct observations of the structure and reactivity of model carbonate interfaces in contact with aqueous brines, petroleum oil, and synthetic oil mixtures to provide new insights into the intrinsic molecular-scale structures and chemical interactions at these interfaces.

### System of study

Most of the worldwide geological formations hosting oil reservoirs consist of a mixture of carbonate rocks, primarily limestone and dolostone. These rocks are composed mostly of nano- to micro-crystalline calcite and dolomite with inclusions of larger (~ 10 μm) single crystals formed during the diagenesis. We probe the structure of freshly cleaved calcite (104) single-crystal surfaces in contact with a suite of fluids, including natural petroleum oil^32^, synthetic aqueous brines of various ionic strengths and compositions, and synthetic oil mixtures in order to test different conceptual models of carbonate–petroleum interactions. (The compositions of these fluids are shown in Tables 1 and 2, and the sequence of fluid exposures for separate calcite samples is indicated in Table 3). The use of single-crystal calcite as a proxy for the major constituent mineral in a natural carbonate rock^33^ is a significant simplification for understanding enhanced oil extraction given the high morphological and compositional complexity of a natural carbonate rock matrix. Nevertheless, this model system contains all of the major components found in the natural system, and enables in situ, molecular-scale observations to reveal the intrinsic interfacial interactions and processes that cannot be obtained in any other way. Ongoing work will connect these observations to the macroscopic behavior observed in natural carbonate rocks^34^.

**Table 1:** Composition and ionic strength of the aqueous brines used in these studies.

Component	Formation water (FW) (ppm)	High salinity water (HSW) (ppm)	Low salinity water (LSW) (ppm)
Sodium, Na^+^	59,491	18,240	1824
Calcium, Ca^2+^	19,040	650	65
Magnesium, Mg^2+^	2439	2110	211
Sulfate, SO_4_^2−^	350	4290	429
Chloride, Cl^−^	132,060	32,200	3220
Bicarbonate, HCO_3_^−^	354	120	12
Total dissolved solids	213,734	57,610	5761
ionic strength (mol/liter)	4.32	1.15	0.12

**Table 2:** Composition of the synthetic oils used in this study (in units of mass fraction).

Component	Dodecane (%)	Synthetic oil mixture
n-Dodecane	100	83.2%
Toluene	0	11.2%
Asphaltene	0	5.6

**Table 3:** Sequence of solutions exposed to each of four separately and freshly prepared calcite samples (solution compositions are listed in Tables 1 and 2). Derived structural parameters describing the calcite–fluid interface for brines, petroleum oil, and synthetic oil mixtures, along with the quality of fit for each measurement.

Sample #	Fluid	<|∆_z_Ca|>		<|∆_z_CO_3_|>		<|∆_θ_CO_3_|>		O_ads_		σ_int_	χ^2^
		Å	w/r to HSW	Å	w/r to HSW	Deg (°)	w/r to HSW	WEq	w/r to HSW	Å	
1	High salinity water (HSW)	0.04	–	0.07	–	4.7	–	2.3	–	1.5	6.6
2	Petroleum	0.57	15.9	0.31	4.7	14.2	3.0	10.9	4.7	7.3	2.1
	HSW	0.09	2.5	0.27	4.1	9.6	2.1	8.5	3.7	4.8	1.6
3	Formation water (FW)	0.07	1.9	0.08	1.2	10.4	2.2	2.3	1.0	1.0	6.6
	Petroleum	0.32	8.9	0.19	2.9	23.9	5.1	5.2	2.3	7.0	2.1
	Low salinity water (LSW)	0.11	3.0	0.12	1.8	13.3	2.8	5.6	2.5	5.9	1.6
4	Dodecane	0.08	2.2	0.13	1.9	6.1	1.3	3.4	1.5	0.0	1.9
	Synthetic oil mixture	0.05	1.4	0.17	2.6	5.0	1.1	2.8	1.2	0.0	2.3
	Dodecane	0.08	2.1	0.09	1.4	4.7	1.0	3.3	1.5	2.3	1.2
	Petroleum	0.59	16.5	0.24	3.6	21.7	4.7	8.8	3.8	6.3	1.0
	Synthetic oil mixture	0.30	8.2	0.16	2.4	31.3	6.7	6.8	3.0	5.0	3.2

The columns labeled “w/r to HSW” show the ratio of the derived parameter value with respect to that observed in HSW.

**Figure 1:**
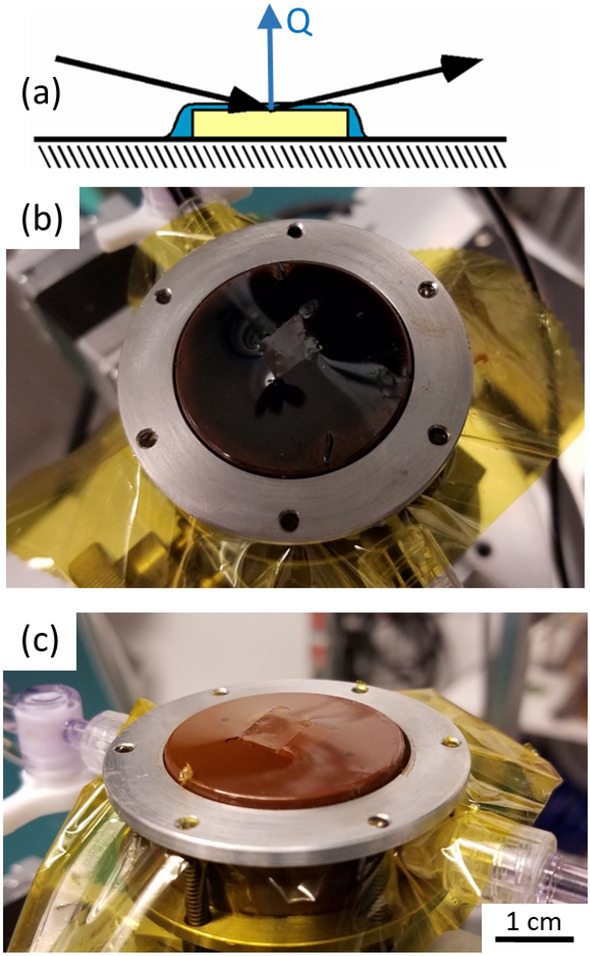
The thin film sample cell used for the XR measurements. (**a**) Schematic of the cell and the specular X-ray scattering geometry (incident and reflected X-rays indicated by black arrows, while the momentum transfer is indicated as a blue arrow). The calcite sample, indicated by the yellow rectangle, is in contact with the fluid (blue) held in place by a Kapton foil (black line). (**b**,**c**) Photographs of the cell filled with petroleum oil and an aqueous brine, respectively.

We probe calcite–fluid interfaces using the technique of X-ray Reflectivity (XR)^35–37^. The specular reflectivity signal (i.e., R(Q), the fraction of the incident X-ray beam that is reflected by the surface) is measured as a function of incident angle, θ, with respect to the surface plane. The scattering condition for each data point is characterized by the “momentum transfer”, Q = (4π/λ)sin(θ), where λ is the wavelength of X-rays, as shown schematically in Fig. 1a. The specular XR signal is due to the interference of X-rays scattered from different molecular layers at the calcite–fluid interface, and its Q-dependent variation has the form of a “crystal truncation rod (CTR)”. The shape of the CTR, especially between the substrate Bragg peaks, is highly interface-sensitive and provides a direct measure of the interfacial structure. Specifically, the XR signals can be calculated, without any adjustable parameters, for any proposed interfacial structure, as defined by a laterally averaged density profile, ρ(z) as a function of height, z, above and below the interface. Consequently the interfacial structure can be determined by least-squares fitting to structural models. (A more complete description of the XR measurement and analysis is included in the “Methods” section, below).

Here, we use a “thin film” cell^37^ in which a calcite sample is held in contact with a thin fluid layer (represented by the blue region in Fig. 1a) with a Kapton membrane. Photographs of the sample cells used for this measurement are shown in Fig. 1b,c. In each image, a calcite sample is at the center, and the ≥ 2 μm-thick fluid film is held in place with a Kapton foil (yellow) that is secured with an aluminum flange. The cell is shown filled with petroleum oil and an aqueous brine in Fig. 1b,c, respectively. Fluids are exchanged in the cell using the fluid ports on the side of the cell body.

The organization of this manuscript is as follows. First we report measurements of freshly prepared calcite surfaces in contact with brine and petroleum to provide a baseline understanding of these interfaces (i.e., without prior exposure to other fluids). Next we show the evolution of the interfacial structure for samples that are equilibrated with aqueous brines before exposure to petroleum, and then subsequently exposed to a brine to simulate the displacement of fluids in a production environment. The comparison of these results for calcite samples with, and without, previous exposure to a brine allows us to constrain the role of aqueous wetting layers separating the calcite surface from the petroleum. Finally, we present results from a calcite sample reacted with synthetic oil mixtures to explore the possible role of the different petroleum components in controlling the observed behavior.

## Results

### Calcite–brine and –petroleum interfaces

**Figure 2:**
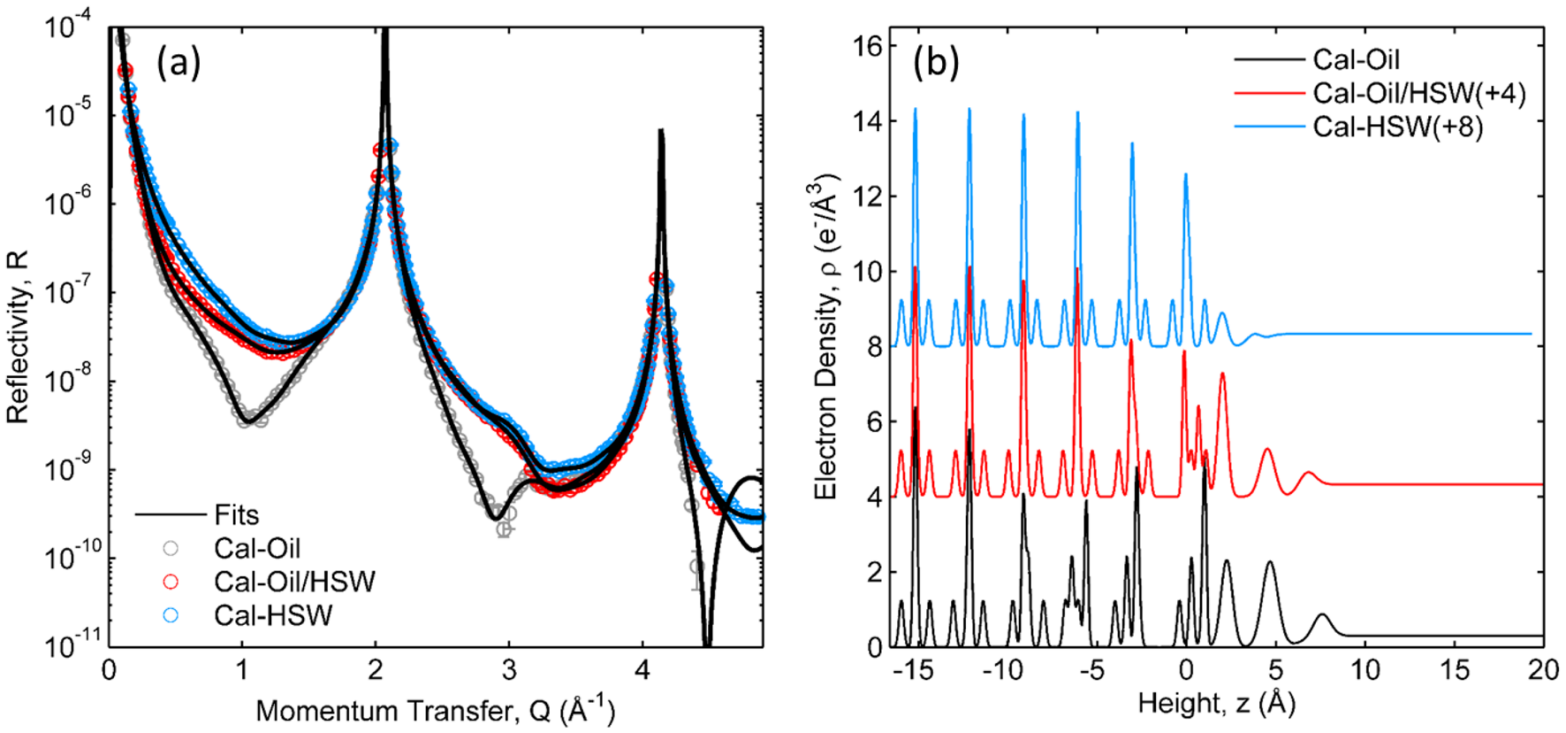
(**a**) X-ray reflectivity data (circles) and model fits (lines) of calcite in contact with high salinity water (HSW, blue), petroleum oil (grey), and in HSW after displacing petroleum oil (red). (**b**) Derived interfacial density profiles corresponding to the model fits.

The calcite–brine interface in high salinity water **(**HSW; Sample 1, Table 3) is a benchmark for understanding petroleum–brine–rock interactions. The XR data show the characteristic crystal truncation rod shape (CTR; Fig. 2a), including the (104) and (208) reflections at Q = 2.07 and 4.14 Å^−1^, respectively, each of which has a peak reflectivity of R ~ 1. The data for the calcite–HSW interface (Fig. 2a, “Cal–HSW”, Sample 1 in Table 3) are visibly similar to previous results of calcite in contact with a calcite saturated solution (“CSS”, i.e., deionized water that is equilibrated with calcite powder and the atmosphere)^25,38^, as are their optimized interfacial density profiles (Fig. 2b, “Cal–HSW”). The derived interfacial structure in HSW includes small but significant changes to the Ca and CO_3_ vertical locations with respect to the bulk calcite structure in the top few CaCO_3_ layers (i.e., for z < 1 Å) and the presence of an interfacial hydration layer^25,38–40^ (i.e., water whose structure is distinct from that of bulk water, analogous to the solvation shell surrounding ions in aqueous solutions). In order to make comparisons of these results to the calcite–petroleum interface, we report characteristic values of the interfacial structure: the average vertical atomic shift magnitudes of Ca and CO_3_ within the top three calcite layers (e.g., <|∆_z_Ca|> = 0.04 Å and <|∆_z_CO_3_|> = 0.07 Å, respectively), the average carbonate tilt angle magnitude in the first three layers with respect to the bulk value (<|∆_θ_CO_3_|> = 4.7°), and the number of water molecules in the primary surface hydration layer per unit cell area of calcite (104) (ΣO_i_ = 2.3). These data are also sensitive to the calcite surface roughness, e.g., due to the presence of topographic steps, which is quantified in terms of a root mean square (rms) variation in its height (σ_int_)^41^. The calcite–HSW interface is found to have only minimal interfacial roughness, σ_int_ = 1.5 Å (compared to the vertical layer spacing of the calcite lattice, d_104_ = 3.035 Å). This value is similar in magnitude to what was observed previously in CSS^25,38^.

The XR data for a freshly cleaved calcite surface in contact with natural petroleum oil (Fig. 2a, “Cal–Oil”; Sample 2 in Table 3) provide insights into the intrinsic carbonate–petroleum interactions. These data show significant differences with respect to calcite in HSW that include large (> 10-fold) reductions in the XR signal near Q = 1 Å^−1^ and 2.8 Å^−1^. These differences are understood using the same structure model used for the calcite–brine interface to quantitatively reproduce the XR signals. This best-fit model reveals three significant changes in the molecular-scale interfacial structure with respect to that seen in HSW (Fig. 2b, “Cal–Oil”). The substrate atomic structural distortions at the calcite–petroleum interface include average vertical changes with respect to the bulk calcite structure of <|∆_z_Ca|> = 0.57 Å for Ca, <|∆_z_CO_3_|> = 0.31 Å for CO_3_, and a mean carbonate tilt magnitude of <|∆_θ_CO_3_|> = 14.2°. These correspond to 16- and 5-fold increases in the average vertical shifts of Ca and CO_3_ ions, respectively, as well as a 3-fold increase in the average angular tilt of the carbonate groups with respect to those seen in HSW. The fluid side of the interface, modeled as a series of adsorbed layers, is also distinct. In the presence of petroleum, the optimized model has three distinct layers, each with a substantially higher electron density than the bulk fluid. In order to compare these differences directly, we model these layers as water molecules and report their electron densities in water equivalents, WEq (i.e., the number of water molecules that would be needed to reproduce a feature in the electron density profile)^42^. We find a combined occupation factor of 10.9 WEq per unit cell area for the first two adsorbed fluid layers, a ~ 5-fold increase with respect to that observed in brine. The observed electron density is due to the combination of the atomic number of each species, Z, and its number density. This suggests that this interfacial layer does not consist primarily of water, because the observed electron density is significantly higher than that for any known phase of water or ice near ambient temperatures and pressures. The data also reveal a large interfacial roughness, σ_int_ = 7.3 Å, a ~ 4-fold increase with respect to that observed in HSW, suggesting some dissolution or growth of the calcite substrate when placed in contact with petroleum. In summary, these results reveal that the calcite interfacial structure experiences significant differences in the brine vs. petroleum environments and that XR measurements are highly sensitive to these differences.

### Brine → petroleum → brine displacement studies of calcite

**Figure 3:**
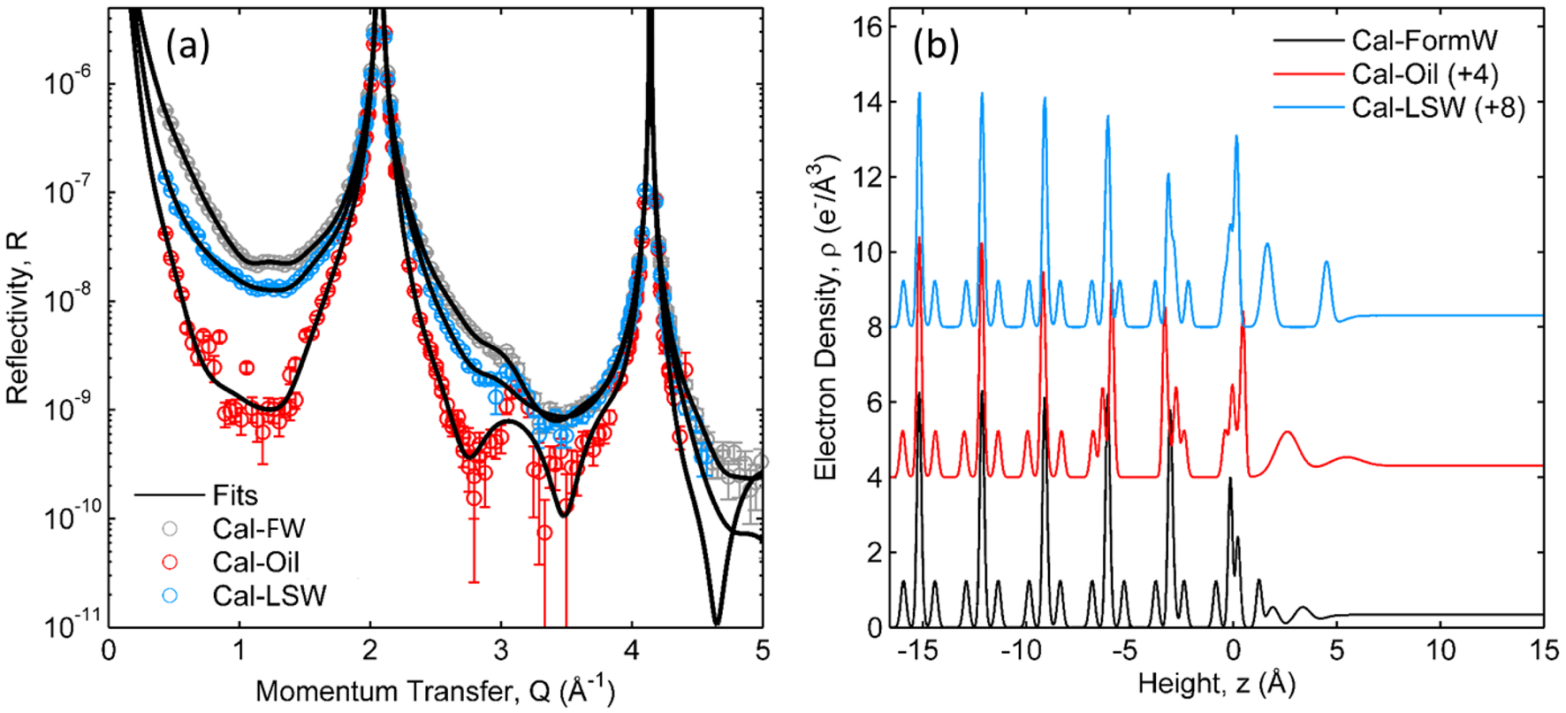
(**a**) X-ray reflectivity data (circles) and model fits (lines) of a calcite in contact with formation water (FW, grey), in petroleum oil after displacement of the FW (red), and in LSW after displacement of petroleum (blue). (**b**) Derived interfacial density profiles corresponding to the model fits.

Similar measurements were performed to understand the changes that occur at the carbonate surface after fluids are displaced. In these measurements, a single calcite surface (Sample 3 in Table 3) was observed sequentially in formation water (FW), in natural petroleum (after displacement of FW) and then in a low salinity water brine (LSW). The XR data and derived interfacial density profile for calcite in FW (Fig. 3a) are similar to those in HSW (Fig. 2) and in CSS from previous studies^25,38^ in spite of their very large differences in ionic strength (Table 1). The XR signals undergo large changes when the FW brine is displaced by petroleum (Fig. 3a, “Cal–Oil”). These XR signals, and the derived structural model (Fig. 3b, Table 3), closely resemble those observed for calcite in petroleum without any prior exposure to a brine (Fig. 2b, “Cal–Oil”; Table 3). Finally, the petroleum was displaced by LSW and the XR signals and derived structural model for the calcite–LSW interface were found to be distinct from those seen for calcite in either petroleum or FW (Fig. 3, “Cal–LSW”).

The differences in the interfacial structures are quantified for each fluid, as described above. In FW, the mean atomic shift magnitudes of the Ca and CO_3_ ions within the top three calcite unit-cell layers are found to be <|∆_z_Ca|> = 0.07 Å and <|∆_z_CO_3_|> = 0.08 Å, with an average carbonate tilt, <|∆_θ_CO_3_|> = 10.4°. In contrast, the same surface after the FW was displaced by petroleum had mean atomic shifts of <|∆_z_Ca|> = 0.32 Å and <|∆_z_CO_3_|> = 0.19 Å, respectively, with an average carbonate tilt, <|∆_θ_CO_3_|> = 24°. We also find that the calcite surface roughness exhibited significant changes, from σ_int_ = 1 Å in FW, to σ_int_ = 7 Å in petroleum. The first two fluid layers have 2.3 WEq in FW, which increases to 5.2 WEq in petroleum.

Additional changes are observed after the displacement of petroleum by the LSW brine (Fig. 3, Table 3). The large structural shifts observed in petroleum were reduced upon the displacement of the petroleum with LSW, where we find mean atomic shifts of <|∆_z_Ca|> = 0.11 Å, <|∆_z_CO_3_|> = 0.12 Å and <|∆_θ_CO_3_|> = 13°. These structural parameters of calcite are more similar to those of calcite in water (e.g., HSW, FW, and CSS). In contrast, we find that the occupation factor for two adsorbed layers (5.2 WEq) and the surface roughness (σ_int_ = 5.9 Å) are very similar to those observed in petroleum. That is, the calcite surface in LSW brine after displacement of petroleum has mixed characteristics: structural changes within the calcite are similar to those seen in water while the electron density of the adsorbed layers and the calcite surface roughness are similar to those seen in petroleum. A similar mixture of characteristics also was observed after displacing the petroleum with HSW (Fig. 2, Table 3).

From these results, we can therefore reach the following tentative conclusions.

- *The calcite–petroleum interface is very similar whether or not there was prior exposure to an aqueous brine. In contrast, the calcite–brine interface shows strong dependence on whether or not the system was pre-exposed to petroleum.* This suggests that calcite is inherently oil-wet.
- *A strong similarity is observed for calcite surfaces in contact with the FW and HSW brines* (from the current study) *and CSS* (from previous work)^38,40^, suggesting that adsorption of ions at this surface (if any) is weak and that the net charge of the calcite surface is small.

### Calcite in synthetic oil mixtures

One of the challenges raised by the above results is to interpret the structural properties of the calcite–petroleum interface. While the XR measurements provide strong constraints on the electron density and thickness of the adsorbed interfacial layer, they do not define its chemical composition. For example, we can conclude that this interfacial layer does not consist primarily of aliphatic hydrocarbon molecules, since the observed electron density is ~ 2- to 4-fold higher than that of pure alkanes. The data, above, suggest that these adsorbed layers derive from petroleum, either by *adsorption* of a petroleum component to the calcite surface or by *reaction* of the calcite surface with the petroleum component.

Given the high compositional and chemical complexity of petroleum oil, additional constraints into the source of these interfacial behaviors can be obtained by comparing these results with that of calcite in synthetic mixtures of pure compounds that contain the major components of natural petroleum. Guiding this work was an initial working hypothesis that the high density layer might be due to the adsorption of organic functional groups that have an intrinsically higher electron density than aliphatic carbon. We chose two synthetic oils to test this idea. The first was n-dodecane as a representative of the aliphatic component of petroleum. We also used a synthetic oil mixture that included dodecane, toluene and asphaltene, as representative of the aromatic and high molecular-weight compounds in petroleum. This mixture is used to see if the presence of structurally denser aromatic rings in the asphaltene^43^ (i.e., in comparison to the aliphatic components) may be responsible for the high electron density layers on the calcite surface.

**Figure 4:**
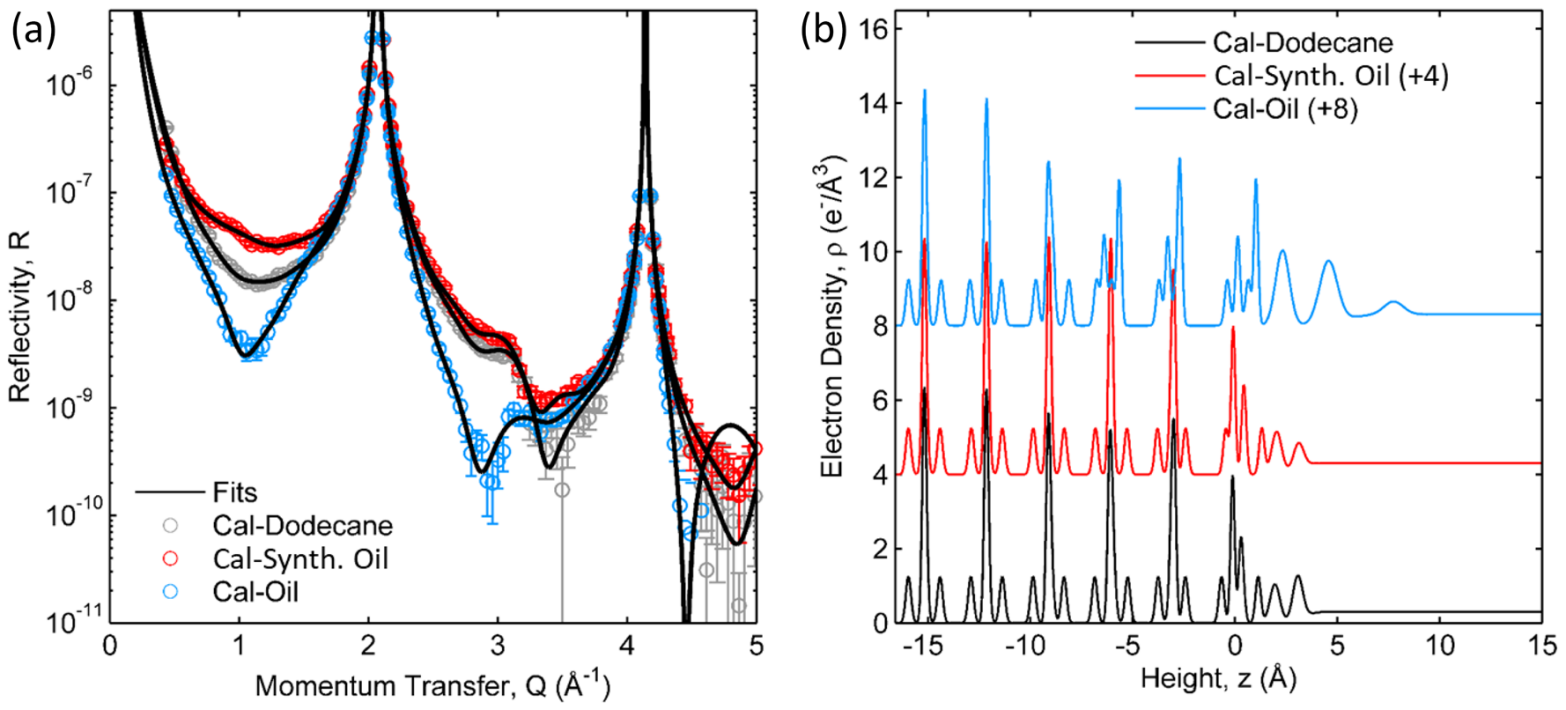
(**a**) X-ray reflectivity data (circles) and model fits (lines) of a calcite in contact with dodecane (grey), the synthetic oil (i.e., dodecane/toluene/asphaltene) mixture after displacement of dodecane (red), and in petroleum oil after displacement of the synthetic oil mixture by dodecane and petroleum (blue). (**b**) Derived interfacial density profiles corresponding to the model fits.

The XR of the calcite–dodecane interface (Fig. 4a) is structurally similar to that observed in the aqueous phases (e.g., HSW and LSW). Notably, the calcite surface is found to have no measurable roughness (consistent with its insolubility in dodecane). The interfacial atomic shifts are small (average structural changes of <|∆_z_Ca|> = 0.08 Å, <|∆_z_CO_3_|> = 0.13 Å, and <|∆_θ_CO_3_|> = 6.1°), and the adsorbed interfacial layer is consistent with a dense “lying down” two-dimensional layer of dodecane (Fig. 4b). That is, none of the characteristics of the calcite–petroleum interface are reproduced in dodecane, even though it represents the major aliphatic component of petroleum.

The same surface was then exposed to a synthetic oil mixture that incorporates toluene and asphaltene components in natural petroleum (i.e., a mixture by weight of 83% dodecane, 11% toluene, and 6% asphaltene) (Fig. 4a). The XR data measured at this interface are different from those observed in pure dodecane. Most notable is the ~ 2-fold *increase* in the XR signal near Q = 1 Å^−1^ in the dodecane/toluene/asphaltene mixture, which is distinct from the ~ 10-fold *decrease* observed in petroleum (Figs. 2 and 3). The optimized structural model that reproduces this behavior (Fig. 4b) is generally similar to that seen in pure dodecane, with no measurable surface roughness (σ_int_ = 0), small surface structural changes (with <|∆_z_Ca|> = 0.05 Å <|∆_z_CO_3_|> = 0.17 Å, and <|∆_θ_CO_3_|> = 5.0°, and an adsorbed layer density that is slightly smaller than that seen in dodecane. These changes observed in the synthetic oil mixture were reversible upon displacement of the synthetic oil mixture by dodecane, in terms of both the XR data and the associated optimized model that has an interfacial structure consistent with that initially observed for the calcite–dodecane interface. That is, adsorption of some components (e.g., toluene or asphaltene) from the synthetic oil mixture to the calcite interface is observed, but it appears to be fully reversible under these conditions and inconsistent to that observed in petroleum.

When the same sample was then exposed to natural petroleum, the XR data showed significant changes including the notable decrease in XR signal near Q = 1 Å^−1^ and 2.9 Å^−1^. The data and optimized model (Fig. 4b) have characteristics that are very similar to those observed for calcite in petroleum (Figs. 2 and 3, Table 3). That is, the surface has roughened substantially (σ_int_ = 6.3 Å), the surface atomic shift magnitudes are large (<|∆_z_Ca|> = 0.6 Å, <|∆_z_CO_3_|> = 0.24 Å, and <|∆_θ_CO_3_|> = 22°), along with a > 2-fold increase in the density of the adsorbed layer. Finally, the same sample was then exposed to the synthetic oil mixture, and the structural characteristics associated with calcite–petroleum interfaces were mostly retained. This result indicates that natural petroleum is able to effectively displace the synthetic oil mixture and that the specific chemical interactions and structures that are responsible for the calcite-petroleum interfacial structure are effectively irreversible and due specifically to the interaction of calcite with the petroleum.

## Discussion

These results reveal a number of insights concerning the interfacial structure of petroleum oil and brines interacting with the calcite (104) surface.

### Calcite–petroleum interfaces

The calcite–petroleum interface exhibits multiple distinct characteristics, including: (1) the formation of a high-density adsorbed layer; (2) the incorporation of significant structural distortions within the top few layers; and (3) an increase in the surface roughness (implying dissolution or growth). These behaviors were observed whenever the calcite surface was in contact with petroleum oil, independent of whether it was a freshly cleaved surface or had previously been exposed to any of the aqueous brines described here (e.g., FW or LSW), or even the synthetic oil mixture. Also the adsorbed layer observed in petroleum oil was not dissolved into the synthetic oil mixture once it formed. The large magnitude of the calcium carbonate interfacial distortions and the effectively irreversible adsorption of the adsorbed layer can be thought of as a “fingerprint” of the intrinsically strong calcite–petroleum interactions. These observations suggest that the properties of this interface are controlled by a component of the petroleum that is strongly attached to the calcite surface (e.g., either by simple adsorption to the surface or by reaction with the calcite to form an interfacial complex). We saw no evidence for a thin water wetting film separating the petroleum from the calcite surface (this is further discussed, below) implying that the pristine calcite surface is intrinsically “oil-wet”.

A primary characteristic of the observed interfacial layer is that its electron density is too large to be attributed to that of a residual thin film of water. The adsorbed layer is present for samples without any prior exposure to brine. This immediately implies that this layer derives primarily from the interaction of calcite with petroleum. The composition or identity of the adsorbed surface film is not uniquely determined by the XR results. However, the data analyses provide some constraints. The high electron density of the adsorbed layers (~ 3–5 times higher than that of water) cannot be explained by adsorption of simple aliphatic molecules (e.g., dodecane or carboxylic acids such as stearic acids) from petroleum oil, as they have electron densities that are typically similar to or *smaller* than water. Our measurements with the synthetic oil mixture was designed to test whether this could be explained by the adsorption of components of petroleum that contain aromatic groups (e.g., asphaltene). The inclusion of this species in the synthetic oil mixture led to behavior that was opposite to that seen in petroleum oil (in terms of the interfacial structure) and its interaction with calcite was reversible (unlike the irreversible formation of an interfacial layer seen in petroleum). These results, therefore, suggest that the observed adsorbed layer at the calcite surface seen in petroleum is not due to the components included in the synthetic oil mixture. However, the behavior could be sensitive to the specific composition of the synthetic oil mixture, especially with respect to saturated, aromatic, resin and asphaltene (SARA) fractions, and further work will be needed for a definitive conclusion.

Another possibility to explain the high electron density of the interfacial layer is that it may be the result of a reaction of calcite with a petroleum component resulting in an interfacial complex. In this scheme, the incorporation of elements (e.g., Ca) having a higher atomic number (Z) would increase the electron density of this interfacial layer. The systematic increase in the observed roughness of the calcite surface after contact with petroleum demonstrates that the morphology of the surface is altered when the calcite is exposed to the petroleum. This increase in roughness is due to changes in calcite surface topography in the form of a laterally variable surface height (e.g., crystallographic steps) although the XR do not distinguish between dissolution and growth. A possible mechanism for the surface roughening is the dissolution of ions from the calcite surface through strong chelation with one or more components in the petroleum. This is fully consistent with strong calcite–petroleum interactions between the adsorbed layer and the calcite surface. It is also consistent with the high electron density of the interfacial layer, as the inclusion of ions in this layer from the dissolution of calcite would increase its electron density with respect to the molecular components of petroleum.

The differences in atomic structural distortions within the calcite surface can be interpreted from a crystal chemistry perspective. Creation of the calcite surface requires that the top layer Ca and CO_3_ ions each lose one Ca–O bond in its coordination shell. The structural distortions within the calcite surface can be used as a measure of the structural and chemical perturbations at the interface in response to this change in the surface coordination environment. In the case of the calcite–water interface, the adsorption of water molecules to the surface Ca and CO_3_ ions completes the coordination shell of these groups and the interfacial structural displacements are generally small^25,38^. In the absence of water, computational studies show significantly larger structural distortions^44^. The structural changes seen for calcite in contact with petroleum oil are even larger than those predicted for a bare calcite surface, suggesting the presence of strong interactions between the calcite surface and a surface-active component that we infer derives from petroleum.

Together, these results, including the structural distortions of the calcite surface, the presence of an adsorbed layer, and surface roughening, suggest that the adsorbed layer modifies the intrinsic calcite surface. It therefore appears likely that this adsorbed layer and its interaction with the petroleum oil will also strongly influence the petroleum wettability of the surface. This interpretation is generally consistent with the conceptual picture of carbonate surface reactivity as the primary control over carbonate wettability in petroleum^16,27–30^, through a combination of surface adsorption of a petroleum component and its complexation with ions from the calcite surface due to carbonate dissolution.

### Calcite–brine interfaces

The calcite surface structures observed in contact with various brines (including formation water, and high- and low-salinity water brines) are essentially similar to that observed previously in calcite-saturated solution^25,38^ in terms of the characteristics of the surface hydration layer, minimal surface roughness, and small structural distortions of calcite. These similarities suggest that there is little or no adsorption of the primary solute ions in these brines (Table 1) to the calcite surface. This is consistent with recent XR measurements and computational simulations showing that the interaction of ions with the calcite–electrolyte interface is either very weak or negligible^26,45^.

### Presence of a water wetting layer

We do not see any evidence for the presence of an aqueous brine film at the calcite–petroleum interface^2–4,13,14,19,20^. This conclusion derives from two observations. First, the calcite–petroleum interface is essentially similar whether or not the calcite surface was previously exposed to a brine before exposure to petroleum. Given the high sensitivity of XR to interfacial structure, this implies that little or no water is present at the calcite–petroleum interface. The second line of evidence derives from what was *not* observed. Thin film structures are well-studied using XR and lead to a periodic oscillation in the reflectivity signal as a function of Q^46,47^ due to the interference between X-rays that are reflected by the top and bottom of the film. For the case of a film of thickness, L, having a uniform density, ρ, the XR signal will exhibit an oscillation of period, ∆Q = 2π/L, where the magnitude of the intensity oscillation is controlled by factors such as the film density (relative to that of the substrate and fluid) and the interfacial roughnesses. Such wetting layers are inferred to have thicknesses in the range of ~ 10–100 Å^2–4^ which would correspond to intensity oscillations with a period of ∆Q ~ 0.6 to 0.06 Å^−1^ in the XR data. This is not observed in any of the XR data (e.g., Figs. 2 and 3) for multiple different brine compositions. Instead, the only evidence for an interfacial “film” is the large dips in reflectivity near Q = 1 and 3 Å^−1^, suggesting ∆Q = 2 Å^−1^, and a film thickness of ~ 3 Å. This, instead, corresponds to the high-density adsorbed layer, described above.

In other words, the XR data show no evidence for a distinct water wetting layer at the calcite–petroleum interface after the brine is displaced by petroleum (although the presence of water as a minor component cannot be excluded). These results are in conflict with two underlying concepts of double-layer expansion (DLE) as the primary mechanism for mediating carbonate–petroleum interactions (e.g., through the presence of a thin water wetting layer whose thickness might be controlled by interactions between charged calcite-water and water–oil interfaces^2–4^).

### Petroleum displacement by brines

The displacement of petroleum oil by an aqueous brine (either the HSW or LSW) leads to a calcite–fluid interface whose characteristics are intermediate between those observed in water and natural petroleum. While the presence of high electron density layers and high surface roughness is typical of that seen at the calcite–petroleum interface, this interface has smaller structural distortions that are more similar to that seen at the calcite–brine interface. This immediately implies that the brine does not effectively displace the interfacial components seen in the petroleum phase.

The large calcite surface structural distortions and high surface roughness that are observed in petroleum are reduced after the petroleum is displaced by the brine, but are still larger than those observed in the brine without prior exposure to petroleum. At the same time, the observed electron density of the adsorbed layer does not change significantly after the displacement of oil by either LSW or HSW brines. From the context of interfacial crystal chemistry discussed above, the reduction in the interfacial structural distortions that were observed after the petroleum was displaced by LSW suggests that water may be able to penetrate into this layer and thereby reduce the chemical stresses imposed by the adsorbed layer through its interaction with calcite. At the same time, the adsorbed interfacial layer was not displaced by the brine, as the enhanced electron density of this layer was similar to that found for measurements of petroleum.

### Synthetic oil mixtures

Synthetic mixtures of the primary components of petroleum oil (i.e., either dodecane or a dodecane/toluene/asphaltene mixture) were used as a proxy for natural petroleum to guide and constrain our interpretation of the observed behaviors. Measurements of the calcite surface in the mixture did not reproduce any of the primary characteristics observed for calcite in natural petroleum. The small but reversible changes observed for calcite in dodecane vs. dodecane/toluene/asphaltene mixtures imply that toluene and/or asphaltene may be surface active species, but they interact weakly with the calcite surface and are readily displaced by dodecane. These results suggest that the observed interfacial behavior of calcite in petroleum is not associated with the primary petroleum components included in the synthetic oil mixture.

### Relevance for oil production

The present results suggest that the *intrinsic* carbonate-petroleum reactions are best described by a conceptual picture that is based on carbonate surface reactivity^16,27–30^ rather than double-layer expansion (DLE)^2–4,13,14,19,20^. That is, our results directly reveal the presence of an interfacial layer that is seen only after interaction of calcite with petroleum, which is formed whether or not the calcite was previously in contact with an aqueous brine, and which is not displaced either by brines or the synthetic oil mixtures. These results suggest that this interfacial layer may play an important role in the wettability of the carbonate mineral surface by petroleum, and presumably the displacement of oil from the surface.

More work needs to be done to connect the observations that are obtained using model systems to the phenomenon of enhanced oil extraction. For example, the present results do not incorporate the high structural and morphological complexity of the natural carbonate rock. Differences in behavior could derive from a number of factors including geometrical factors (confinement and curvature of rock pores), mineralogy (e.g., calcite vs. dolomite), or interfacial crystal chemistry (e.g., different crystallographic orientations of a given mineral phase). Consequently, additional measurements will be needed to test the applicability of the insights from these model systems to the production environment. Additional work will also need to identify the composition and reactivity of the observed adsorbed interface layers, and how these characteristics depend on the brine composition. Finally, additional studies will need to discern the role of injection brines in controlling rock wettability and oil displacement in natural carbonate rock samples to understand whether those behaviors are comparable to that seen here on single-crystal calcite surfaces.

## Summary

The present results provide new molecular-scale insights into the intrinsic interactions and structures that occur at the interface between calcium carbonate (i.e., calcite) and the fluids associated with oil production, including natural petroleum oil, aqueous brines (including formation water, high and low salinity water), and synthetic oil mixtures. These results show that the intrinsic interactions between calcite and petroleum are better described as an “oil-wet” instead of “water-wet” interface. The properties of this interface appear to be controlled by the presence of an interfacial layer (e.g., likely formed by adsorption of a surface active component from petroleum, and possibly its complexation or chelation with calcium carbonate). This interfacial species appears to be strongly bound since it is not displaced by either the aqueous brines or the synthetic oil mixture. However, its composition is, as of yet, undefined. Additional features of this interface, especially when compared to the calcite–brine interfaces, include significant structural distortions within the top few calcium carbonate layers, and a significant surface roughness. That is, the results reveal that the molecular-scale structure and interactions of carbonate surfaces with natural petroleum and brines are distinct and that these differences are likely to be key to establishing the fundamental chemical controls over its wettability.

## Methods

### Sample preparation and materials

The single crystal calcite surfaces were prepared as described previously^40^. Briefly, rods of calcite were cut with diamond saw from a large (~ 1–2 in.) sized calcite crystal, with the rods having a cross section of ~ 5 mm × ~ 10 mm, with the axis of the rod oriented orthogonal to the calcite cleavage plane. The calcite surfaces were then created by cleaving the calcite crystal with a razor blade, by tapping with a hammer, exposing a clean calcite (104) surface.

The aqueous brine solutions are similar to those used previously^7,9,32^. The compositions of these solutions are shown in Table 1. The solutions were mixed from pure salt compounds (e.g., Na_2_SO_4_, NaHCO_3_, NaCl, CaCl_2_, CaCl_2_·2H_2_O, MgCl_2_·6H_2_O). The natural petroleum oil was obtained from a carbonate reservoir, and its composition was previously characterized^32^. The synthetic oil mixture (with a composition indicated in Table 2) was created by mixing n-dodecane with a 2:1 mixture of toluene with asphaltene resulting in a mass fractions of 83% dodecane, 11% toluene and 6% asphaltene. The asphaltene compound was obtained from crude oil through solvent extraction using ASTM D6560 protocol^48^.

### X-ray reflectivity

The ability to probe solid–liquid interfaces through direct in-situ observations can extremely challenging due to the inability of most interface-sensitive probes (e.g., typically electron based spectroscopies^49^) to penetrate fluid layers when the fluid thickness exceeds even a few nm’s. The availability of experimental probes such as synchrotron X-ray scattering has enabled a revolution in our understanding of solid–water interfaces^22,39,50^. Such understanding obtained by the use of single crystal surfaces, provides an avenue to better comprehend the intrinsic fluid–solid interactions at the molecular-scale. In particular, substantial work has been done to understand the structure and reactivity of the calcite(104)–water interface which is flat, free from contaminants, and has a unique termination^25^.

Probing the interaction of carbonates with petroleum presents additional challenges, including the high compositional complexity of petroleum oil^7,9,32^ and its optical opacity. These challenges can be met using well-established approach of X-ray reflectivity (XR) to understand the intrinsic structure of carbonate interfaces in contact with natural and synthetic petroleum oils and brines. The penetration of X-rays through petroleum is similar to that of water and so the XR capabilities previously developed to understand mineral–water interfaces can be applied directly to the structure and composition of carbonate–petroleum interfaces.

Briefly, the XR technique^35–37^ probes interfacial structure through the variation of the specular (i.e., mirror-like) reflected intensity, I_R_, as a function of the angle of incidence (θ, with respect to the surface plane). It is convenient to recast this information in terms of the interfacial reflectivity, R(Q) = I_R_(Q)/I_0_, as a function of the “momentum transfer”, Q = (4π/λ)sin(θ), where I_0_ is the incident beam intensity. In particular, the measured XR signal, R(Q), is related directly to the laterally averaged electron density profile at the solid–liquid interface, ρ(z), as a function of the height, z, above and below the interface through the relation:

$${\text{R}}\left( {\text{Q}} \right) = ({4}\pi {\text{r}}_{{\text{e}}} /{\text{A}}_{{{\text{UC}}}} {\text{Q}})^{{2}} \left| {\int {\rho \left( {\text{z}} \right){\text{ exp}}\left( {{\text{iQz}}} \right){\text{dz }}} } \right|^{{2}} ,$$

where r_e_ = 2.818 × 10^–5^ Å, and A_UC_ = 20.2 Å^2^ is the surface unit cell area of the calcite(104) surface. That is, the reflectivity signal is related to the Fourier transform of the electron density across the interface. This simple and direct relationship between the unknown structure and the measured signal allows models of the interface to be quantitatively tested and optimized to reveal the interfacial structure. Specifically, the interfacial structure is obtained through least-squares fitting of the data using molecular-scale models that include the mineral surface structure, the presence of any adsorbed species from the fluid, and the average surface roughness. The models ultimately specify the height, z_i_, occupation, O_i_, and root-mean square distribution, σ_i_, for each atom, i, at the interface. In many cases, we make use of well-established chemical constraints, such as the description of the carbonate group as a rigid object defined by its height and its rotation. This is described by a tilt angle, θ, (for tilts of the carbonate ion plane with respect to the physical surface plane) and a twist angle, ϕ, (for rotations around the axis normal to the carbonate plane). Within this picture, the reflectivity signal can be written as:

$${\text{R}}\left( {\text{Q}} \right) = ({4}\pi {\text{r}}_{{\text{e}}} /{\text{A}}_{{{\text{UC}}}} {\text{Q}})^{{2}} \left| {\Sigma_{{\text{j}}} {\text{f}}_{{\text{j}}} \left( {\text{Q}} \right){\text{O}}_{{\text{j}}} {\text{exp}}\left( {{\text{iQz}}_{{\text{j}}} } \right){\text{ exp}}\left[ { - {\raise0.7ex\hbox{$1$} \!\mathord{\left/ {\vphantom {1 2}}\right.\kern-\nulldelimiterspace} \!\lower0.7ex\hbox{$2$}}({\text{Q}\sigma}_{{\text{j}}} )^{{2}} } \right]} \right|^{{2}} ,$$

where the sum is over all atoms, j, in the sample. This picture can be further simplified by separating this sum into three components, of which two are known (i.e., the bulk substrate and fluid layers), so that we can write:

$${\text{R}}\left( {\text{Q}} \right) = ({4}\pi {\text{r}}_{{\text{e}}} /{\text{A}}_{{{\text{UC}}}} {\text{Q}})^{{2}} \left| {{\text{ F}}_{{{\text{sub}}}} \left( {\text{Q}} \right) + {\text{F}}_{{{\text{int}}}} \left( {\text{Q}} \right) + {\text{F}}_{{{\text{fluid}}}} \left( {\text{Q}} \right) \, } \right|^{{2}} .$$

These three terms represent the structure factors, F(Q), of the crystalline substrate, F_sub_(Q), the interfacial region, F_int_(Q), and the fluid above the surface, F_fluid_(Q). In this representation, the contributions from the substrate and fluid are known a priori (and are defined by the known bulk crystal structure of calcite, and the known density of the fluid). The only unknown in the reflectivity signal derives from the structure at the interface, including the top few calcite surface layers and the near-surface adsorbed layers. Previous work has shown that the interfacial structure differs from the bulk structure by depth-dependent atomic layer shifts that are measurable for the top four calcite layers. Similarly, it has been found that the calcite–CSS interface is characterized by two layers of adsorbed water molecules (constituting an interfacial hydration layer) followed by a bulk-like fluid density. The structure of the interfacial fluid layer depends on the chemical interactions at the solid–fluid interface, and can extend as far as ~ 1 nm from the solid surface.

In the case of the petroleum–carbonate interface, it is important to recognize that some caution is needed in interpreting the optimized structural models. The model implicitly assumes that the interfacial structure is laterally uniform (i.e., having the same molecular-scale structure within each surface unit cell) and that it can be described by a unique substrate structural distortion, a few adsorbed species at the calcite surface, along with a bulk-like fluid above the surface. The composition of the petroleum oil, unlike simple brine solutions, is highly complex, with hundreds of individual molecular components, each of which may behave differently. Furthermore, the inferred interfacial structural distortion is described by large structural changes that are likely to be controlled by the specific interaction with adsorbed species that may include large molecules and oligomers. Therefore, the actual interfacial structure is likely to be laterally heterogeneous at the molecular-scale. As such, the derived structural models should not be taken literally (i.e., as indicating the actual location of each atom and molecule), but instead are best interpreted as representing the effective (laterally averaged) interfacial structure. Consequently, our interpretation emphasizes the large-scale trends in the structural models (average atomic shift magnitudes, total density, etc.) to reveal the key characteristics (or, “fingerprints”) of these calcite–fluid interactions.
